# Preparation and Properties of Inherently Black Polyimide Films with Extremely Low Coefficients of Thermal Expansion and Potential Applications for Black Flexible Copper Clad Laminates

**DOI:** 10.3390/polym12030576

**Published:** 2020-03-05

**Authors:** Yao-yao Tan, Yan Zhang, Gang-lan Jiang, Xin-xin Zhi, Xiao Xiao, Lin Wu, Yan-Jiang Jia, Jin-gang Liu, Xiu-min Zhang

**Affiliations:** 1 Beijing Key Laboratory of Materials Utilization of Nonmetallic Minerals and Solid Wastes, National Laboratory of Mineral Materials, School of Materials Science and Technology, China University of Geosciences, Beijing 100083, China; 2003190022@cugb.edu.cn (Y.-y.T.); 2003180029@cugb.edu.cn (G.-l.J.); 2003180031@cugb.edu.cn (X.-x.Z.); 2003190023@cugb.edu.cn (L.W.); 2003190024@cugb.edu.cn (Y.-J.J.); 2 School of Electrical Engineering, Beijing Jiaotong University, Beijing 100044, China

**Keywords:** black polyimide film, coefficient of thermal expansion (CTE), optical properties, thermal properties, flexible copper clad laminates (FCCL)

## Abstract

In the current work, a series of black polyimide (PI) films with excellent thermal and dimensional stability at elevated temperatures were successfully developed. For this purpose, two aromatic diamines including 4,4′-iminodianline (NDA) and 2-(4-aminophenyl)-5- aminobenzimidazole (APBI) were copolymerized with pyromellitic dianhydride (PMDA) to afford PIs containing imino groups (–NH–) in the molecular structures. The referenced PI film, PI-ref, was simultaneously prepared from PMDA and 4,4′-oxydianiline (ODA). The introduction of imino groups endowed the PI films with excellent blackness and opaqueness with the optical transmittance lower than 2% at the wavelength of 600 nm at a thickness of 25 μm and lightness (L^*^) below 10 for the CIE (Commission International Eclairage) Lab optical parameters. Meanwhile, the introduction of rigid benzimidazole units apparently improved the thermal and dimensional stability of the PI films. The PI-d film based on PMDA and mixed diamines (NDA:APBI = 70:30, molar ratio) showed a glass transition temperature (*T*_g_) of 445.5 °C and a coefficient of thermal expansion (CTE) of 8.9 × 10^−6^/K in the temperature range of 50 to 250 °C, respectively. It is obviously superior to those of the PI-a (PMDA-NDA, *T*_g_ = 431.6 °C; CTE = 18.8 × 10^−6^/K) and PI-ref (PMDA-ODA, *T*_g_ = 418.8 °C; CTE: 29.5 × 10^−6^/K) films.

## 1. Introduction

Aromatic polyimide (PI) films represent a class of high-performance polymer films characterized by excellent engineering properties, including extraordinary thermal and extreme cold resistance, good chemical and radiative stability, good electrical and thermal insulating properties, and good flame retardancy [1,2,3]. Their excellent comprehensive properties make PI films good candidates for various high-tech applications. In recent years, with the rapid development of the microelectronic and optoelectronic industry, the applications of PI films in these areas have attracted increasing attention from both academic research and engineering fabrications. For instance, the research on the adjustment of optical transparency and colors of PI films so as to meet the property requirements of high-tech areas has been becoming one of the most vigorous topics in the PI materials [4,5,6]. Standard PI films, characterized by the first commercially available poly(pyromellitic dianhydride-co-4,4′-oxydianline) (PMDA-ODA) commercialized by DuPont company, USA in the 1960s with the trademark of Kapton^®^, usually exhibit a golden yellow color and a certain extent of optical transparency owing to the absorption of visible light caused by the intra- and intermolecular charge transfer behaviors in the polymer chains [7,8,9]. The charge transfer complex (CTC) has been proven to be formed with the diamine unit as the electron donator and the dianhydride unit as the electron acceptor [10,11,12]. Up to now, most of the research has been focused on eliminating or prohibiting the CTC formation in the PI molecular chains so as to afford colorless and transparent PI (CPI) films, which have been highly desired in advanced optoelectronic applications, such as flexible display, solar energy systems, and so on [13,14,15,16]. However, in some applications, PI films with enhanced CTC formation are also desirable due to the specific functionalities of such PI films [17]. Among these PI films, intrinsically black PI films have been widely researched and developed in the past decades.

**Figure 1:**
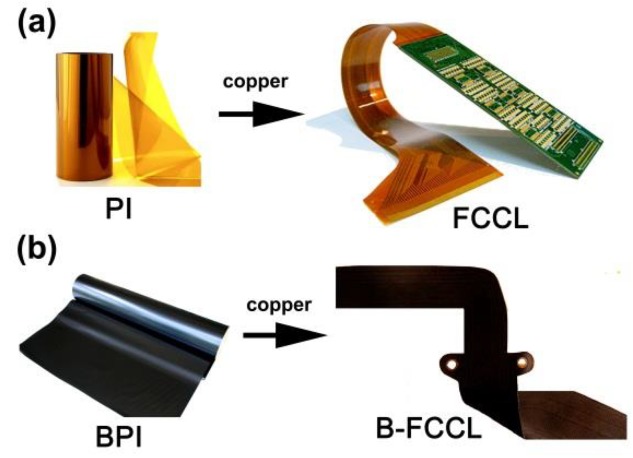
Applications of black polyimide (PI) films in flexible copper clad laminates (FCCLs).

One of the most successful applications for black PI films might be their applications in flexible copper clad laminates (FCCLs), as shown in Figure 1. Undoubtedly, FCCLs are the key components achieving the miniaturization, integration, lightweight, and flexibility of electronic devices [18,19,20]. Generally, conventional FCCLs are made of standard PI films and copper foils, thus exhibiting a yellow appearance, as illustrated by Figure 1a. However, FCCLs fabricated on black PI films (B-FCCL, Figure 1b) are recently desired in the microelectronic industry because the black and opaque PI films prevent the potential degradation of the fabricated circuits caused by the environmental long-term ultraviolet radiation. In addition, the black PI films are often used to cover the lower circuits for intellectual property protection purposes. Besides the technical factors for the applications of black PI films in FCCLs, there are also some aesthetic considerations for the practical applications. Black PI films have also been investigated as high-temperature resistant labels, loudspeakers, and thermal control blankets in civilian and military fields.

Up to now, most of the black PI films have been developed by composite methodology, which is the combination of standard PI films with black additives, either with the inorganic ones, such as carbon black [21,22], carbon nanotube [23], and graphene [24], or with the organic black dyes. As we know, the inorganic black dyes or pigments are usually electrically conductive; thus, the derived black PI composite films usually exhibit reduced electrical resistivities. In some cases, this reduction of electrical properties is desired for practical applications, such as the black PI films designed for the applications in space vehicles in order to remedy the electrostatic discharge issues of the accumulated charges in the standard PI film components caused by their high electrical insulating nature [25]. Again, as in the case of black high-temperature resistant labels, black and electrical conductive additives are often introduced into the PI matrix so as to eliminate the effects of electrostatic charges on the printing process. However, in most of the microelectronic applications, the sacrifice of the electrical and dielectric properties of the black PI film matrix due to the addition of conductive additives is often unfavorable. Thus, intrinsically black PI films with maintained thermal and electrical properties are highly desired in the modern microelectronic applications. 

In our previous work, a series of intrinsically black PI films were developed by the introduction of imino (–NH–) units in the standard PMDA-ODA film via the copolymerization of an aromatic diamine, 4,4′-iminodianiline (NDA) [26]. The imino (–NH–) unit present in the NDA molecular structure can be oxidized in the presence of trace oxygen, resulting in the black color of the PI films. This color change is more significant in the high-temperature oxidative environment [27]. On the other hand, the lone pair electrons on the nitrogen atoms in imino unit are easily attracted toward the electron-deficient carbonyl groups in the dianhydride moiety, leading to an enhanced charge migration and flow in the molecular chains of the NDA-PIs, which is beneficial for the coloration of the NDA-PI films according to the CTC theory. These two factors endowed the NDA-PI films excellent blackness and opaqueness. Meanwhile, another benefit for the NDA-containing PI is the reduction of coefficient of thermal expansion (CTE) of the film. The PI (PMDA-NDA) film showed a CTE value of 18.8 × 10^−6^/K in the temperature range of 50 to 250 °C, which is obviously lower than that of PI (PMDA-ODA) (CTE: 29.5 × 10^−6^/K). This low-CTE feature is quite valuable for the practical applications of NDA-PI films because most of the polymer films exhibited much higher CTE values than those of the common inorganic or metal substrates, such as copper foil (CTE: approximately 17.0 × 10^−6^/K), silica wafer (CTE: approximately 3.0 × 10^−6^/K), indium tin oxide (CTE: approximately 7.2 × 10^−6^/K), and so on. The mismatch of the CTE values of the polymer components and the related inorganic or metal substrates is thought to be one of the most common reasons for the reliability issues of the devices, such as delamination, warpage, cracking, and so on [28]. 

In the current work, in order to further decrease the CTE values of the black NDA-PI films, rigid benzimidazole units were endeavored to be introduced into the molecular chains of the NDA-PIs via a second diamine, 2-(4-aminophenyl)-5-aminobenzimidazole (APBI). The influence of the introduction of benzimidazole units on the thermal and optical properties of the PI films was studied in detail.

## 2. Materials and Methods

### 2.1. Materials

Pyromellitic dianhydride (PMDA) was purchased from Shijiazhuang HOPE Chem. Co. Ltd. (Shijiazhuang, China) and dried at 180 °C in vacuum for 24 h prior to use. APBI was purchased from Changzhou Sunlight Pharmaceutical Co. Ltd. (Changzhou, China) and recrystallized from absolute ethanol and discolored with active charcoal powder before use. *N*-methyl-2-pyrrolidone (NMP), *N*,*N*-dimethylacetamide (DMAc), and other solvents were obtained from Tokyo Chemical Industry Co., Ltd. (Tokyo, Japan) and purified by distillation prior to use. 

4,4′-Iminodianiline (NDA) was synthesized in our laboratory [26] and purified by recrystallization from absolute ethanol under the protection of high-purity nitrogen. Pure NDA diamine was obtained as colorless needles with the purity of 99.5% according to the gas chromatography analysis. It tends to discolor with the exposure of visible light or storage in air, especially staying in good solvents, such as NMP and DMAc. However, it is very stable and can maintain the colorless state for years if it is stored in an inert and dark place.

### 2.2. Characterization

The inherent viscosity of the PI precursors, poly(amic acid) (PAA), was measured using an Ubbelohde viscometer (Mitong Electromechanical Tech. Co. Ltd., Shanghai, China) with a 0.5 g/dL NMP solution at 25 °C. The number average molecular weight (*M_n_*) and weight average molecular weight (*M_w_*) of the poly(amic acid)s (PAAs) were recorded by a gel permeation chromatography (GPC) (Shimadzu, Kyoto, Japan) with the high-pressure liquid chromatography (HPLC) grade of NMP as the mobile phase. The attenuated total reflectance Fourier transform infrared (ATR-FTIR) spectra of the PI films were measured by an Iraffinity-1S FT-IR spectrometer (Shimadzu, Kyoto, Japan) with the scanning range of 4000–400 cm^−1^. Ultraviolet-visible (UV-Vis) spectra were performed on a Hitachi U-3210 spectrophotometer (Tokyo, Japan). Wide-angle X-ray diffraction (XRD) was conducted on a Rigaku D/max-2500 X-ray diffractometer (Tokyo, Japan) with Cu-Kα1 radiation, operated at 40 kV and 200 mA. The CIE (Commission International Eclairage) Lab parameters of the PI films were recorded on an X-rite color i7 spectrophotometer (Grand Rapids, MI, USA) with PI samples at a thickness of 50 μm according to ASTM D1925. 

The thermal properties of the PI films, including thermogravimetric analysis (TGA), differential scanning calorimetry (DSC), dynamic mechanical analysis (DMA), and thermomechanical analysis (TMA) were evaluated with the thermal analysis systems of TA Instruments (New Castle, DE, USA) with the apparatus of Q50, Q100, Q800, and Q400, respectively. 

### 2.3. PI Synthesis and Film Preparation

**Figure 2:**
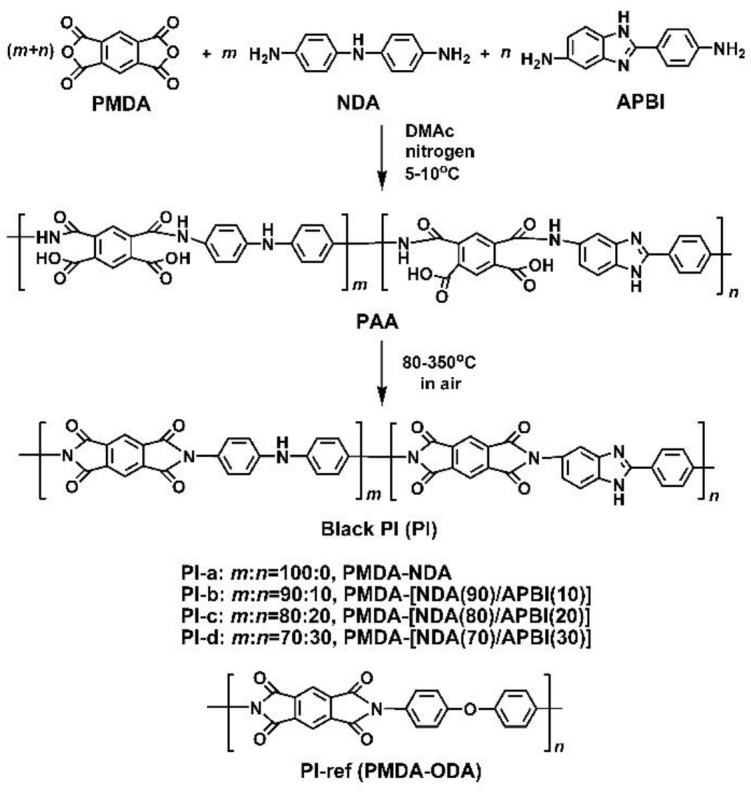
Synthesis of black PIs and PI-ref films.

Five PI films, including two homopolymers, PI-a (PMDA-NDA) and PI-ref (PMDA-ODA), and three copolymers, PI-b, PI-c, and PI-d with the respective NDA/APBI molar ratios of 90:10, 80:20, and 70:30 were successfully prepared with the procedure shown in Figure 2. It can be deduced from the figure that both of the NDA and the APBI diamines contain imino (−NH−) units in their structures, whose difference is that the imino unit is in the main chain for the former and in the imidazole ring for the latter. Thus, it can be expected that the blackness and other color parameters of the NDA-PI films might be maintained for the copolymer films.

The PI films were prepared by a two-step polycondensation procedure. First, the soluble PI precursors, poly(amic acid)s (PAAs) were synthesized, which can be illustrated by the synthesis of PI-d. A polycondensation apparatus was first set up, which was comprised of a 500 mL three- necked glass flask equipped with a mechanical stirrer, a cold-water bath, and a nitrogen inlet. To the apparatus was added newly dried DMAc solvent (100.0 g), and the air in the system was replaced by continuous nitrogen flow. Then, NDA (13.9475 g, 70 mmol) and APBI (6.7278 g, 30 mmol) were added, and the reaction system was cooled to 5–10 °C. A clear diamine solution was obtained after stirring for 10 min under the flow of nitrogen. Then, PMDA (21.8120 g, 100 mmol) was added to the diamine solution together with an additional volume of DMAc (70.0 g). The solid content of the reaction system was controlled to be 20 wt %. The reaction mixture was stirred for 1 h, and then the cold-water bath was removed. The reaction was prolonged for 24 h at room temperature. Then, the obtained viscous deep-color PAA-d solution was filtered through a sintered glass funnel with the filtration accuracy of 10 μm. The purified PAA-d solution was spin-coated on a clean glass substrate. Then, the glass substrate with the PAA-d solution were thermally baking in a high-temperature baking oven with circulation fans by the procedure of 80 °C/3 h, 150 °C/1 h, 180 °C/1 h, 250 °C/1 h, 300 °C/1 h, and 350 °C/1 h. After cooling to room temperature, the glass plate was immersed into deionized water. The self-standing black PI-d film was obtained by peeling off the glass plate. By the similar procedures, the other PI films, including PI-a, PI-b, PI-c, and PI-ref films were also obtained.

## 3. Results and Discussion

### 3.1. PI Synthesis and Film Preparation

First, the effects of the molecular structure on the solution properties and molecular weights of the PAAs were studied, and the data are tabulated in Table 1. It can be seen that the introduction of the APBI component apparently decreased the inherent viscosities and molecular weights of the PAAs. The number average molecular weights (*M*_n_s) of the PAAs decreased with the increasing contents of the APBI in the polymers as follows: PI-a > PI-b > PI-c > PI-d. This might be due to the relatively low reactivity of the APBI diamine, in which the electron densities on the amino groups are decreased via the conjugated pathway along the benzimidazole ring. A similar phenomenon has also been observed in our previous work [29]. Nevertheless, all the PAAs afforded flexible and tough black PI films. 

**Figure 3:**
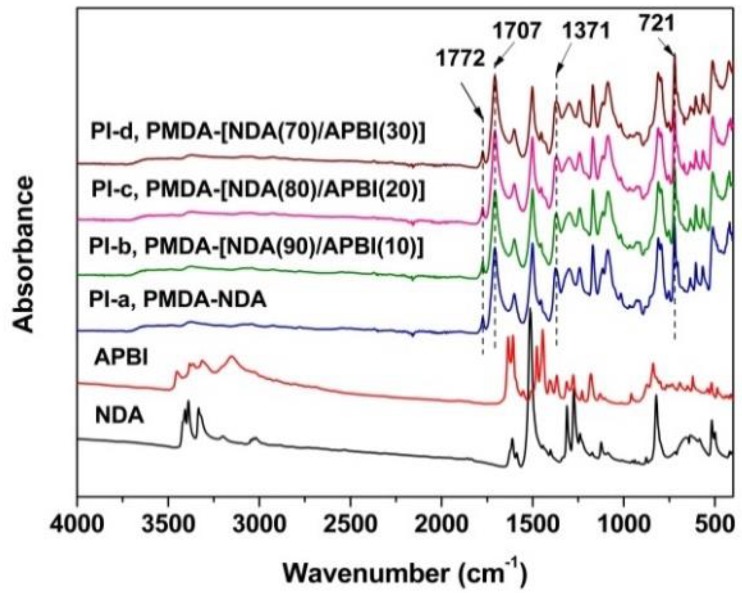
Attenuated total reflectance Fourier transform infrared (ATR-FTIR) spectra of PI films and the starting diamine monomers.

The chemical structures of the PI films were confirmed by the ATR-FTIR measurements, and the results are shown in Figure 3. For comparison, the spectra of the starting NDA and APBI diamines were also presented in the figure. It can be clearly seen that the characteristic absorptions of the amino (−NH_2_) in the diamines around the wavenumber of 3500–3200 cm^−1^ totally disappeared in the spectra of the PI films, indicating the successful conversion from the monomer to the PIs. Instead, the characteristic absorptions of imide rings were all clearly observed, including the absorptions at 1772 cm^−1^ assigned to the asymmetrical carbonyl stretching vibrations, at 1707 cm^−1^ assigned to the symmetrical carbonyl stretching vibrations, at 1371 cm^−1^ for the C–N stretching vibrations, and at 721 cm^−1^ for the carbonyl out-of-plane bending vibrations.

**Figure 4:**
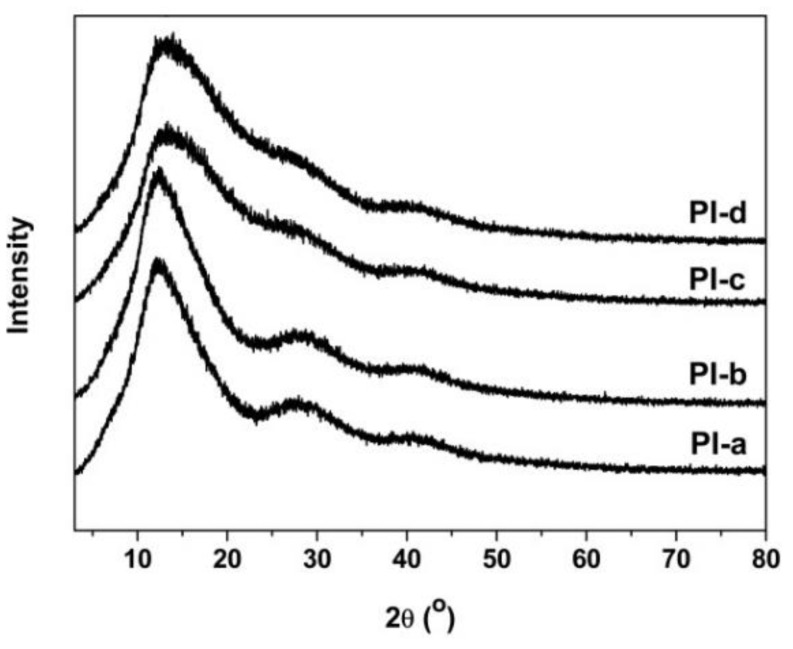
XRD patterns of black PI films.

The microscopic molecular packing states of the PI films were investigated by XRD measurements, and the results are shown in Figure 4. It can be observed that the peaks of scattering angles (2*θ*) of the PI films slightly increased with the increasing APBI contents in the PI films. For instance, the 2*θ* values of the PI films increased with the order of PI-a (12.14°) < PI-b (12.47°) < PI-c (13.14°) < PI-d (13.98°). The PI films exhibited typical amorphous natures. 

### 3.2. Optical Properties

**Figure 5:**
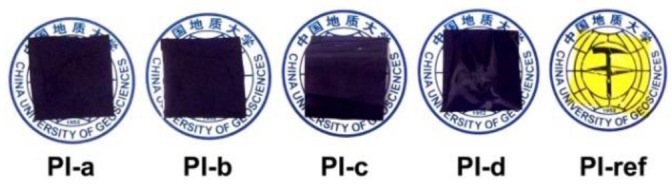
Appearance of black PI and PI-ref films.

**Figure 6:**
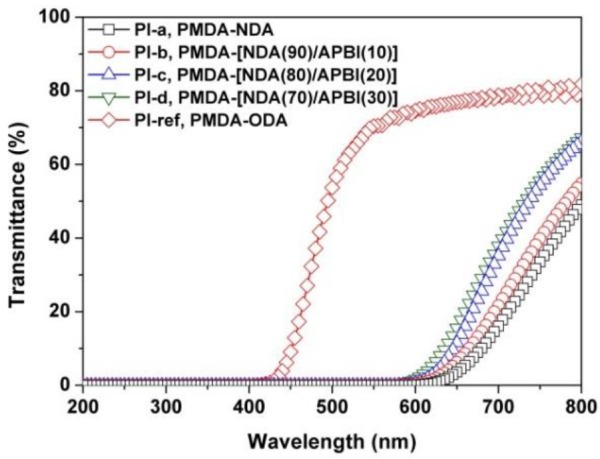
UV-Vis spectra of black PI and PI-ref films.

The influence of the benzimidazole units on the optical properties of the NDA-PI films was studied by optical transmittance and CIE Lab measurements. Figure 5 shows the appearance of the NDA-PI films and the PI-ref film. It can be visually seen that the PI films based on NDA diamine showed a totally black appearance, which is quite different from that of the standard PI-ref film. It is quite difficult to judge the effects of the introduction of benzimidazole units on the color changes of the NDA-PI films with naked eyes. Then, the optical transmittances of the PI films were quantitatively investigated by the UV-Vis measurements, and the results are shown in Figure 6 and Table 2. As can be deduced from the figure, the NDA-PI films with the thickness of 25 μm showed good opaqueness with the cutoff wavelengths (*λ*) of 561–592 nm, which are much higher than that of PI-ref (*λ* = 407 nm). The NDA-PI films showed optical transmittance values below 2% at the wavelength of 600 nm, while the PI-ref showed the value of 74.6% at the same wavelength. At the visible light region of 760 nm, the NDA-PI films also exhibited much lower transmittances compared to the PI-ref. The introduction of benzimidazole units into the NDA-PI films deteriorated the blackness of the NDA-PI films to some extent. For instance, the *T*_760_ values of the PI films increased with the order of PI-a < PI-b < PI-c < PI-d < PI-ref. The *λ* values of the PI films also decreased with the increasing of the benzimidazole contents in the PI films. Nevertheless, the copolymerized PI films still showed a low optical transmittance in the visible light region. 

**Figure 7:**
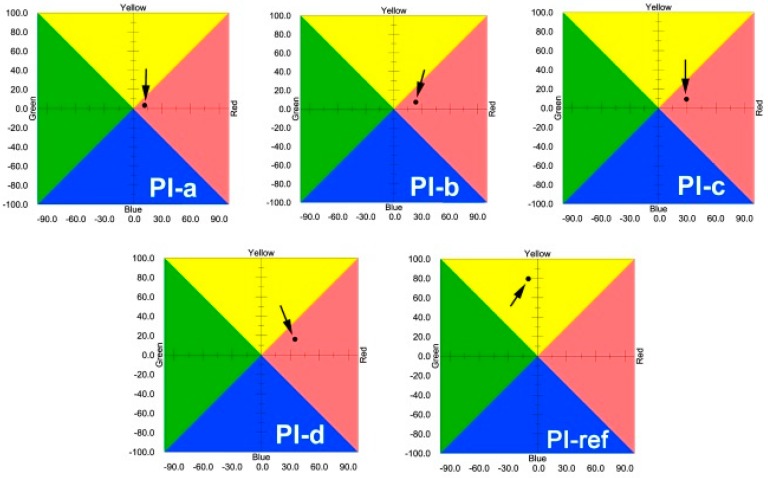
Commission International Eclairage (CIE) Lab color parameters of PI films.

The effects of the chemical structures of the PI films on their optical properties were further investigated by the CIE Lab measurements. Figure 7 and Table 2 depict the optical parameters of the PI films. It can be deduced from the increasing L^*^, a^*^, and b^*^ values of the PI films that the color of the PI films respectively changed toward brightness, redness, and yellowness as the content of benzimidazole increased. Comparatively speaking, the PI-ref film presents the essence of yellow and green nature with a much higher b^*^ value and a negative a^*^ value. This also indicates that the introduction of benzimidazole units is not quite beneficial for endowing the PI films with high blackness and opaqueness. However, the degree of blackness of the PI-a (PMDA-NDA) film was maintained to a great extent when the contents of benzimidazole unit were less than 30% (mole ratio) in the diamine moiety.

### 3.3. Thermal Properties

**Figure 8:**
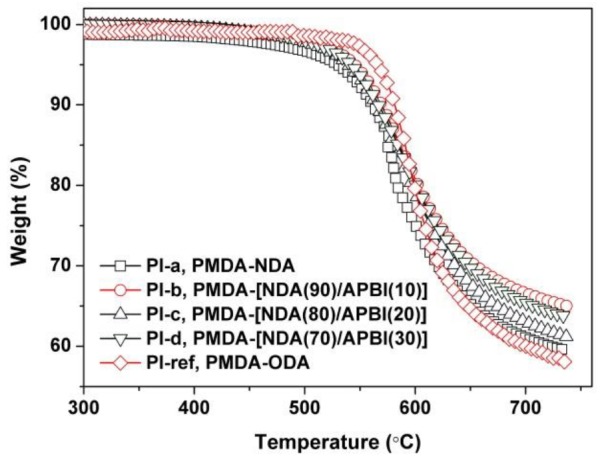
Thermogravimetric analysis (TGA) plots of black PI and PI-ref films in nitrogen.

One of the main purposes of the current work is to further reduce the CTE values of the intrinsically black NDA-PI films via the introduction of benzimidazole units. According to the optical property investigation, most of the blackness and opaqueness of the NDA-PI films was maintained. Then, the thermal properties of the copolymerized PI films were further studied by various measurements, including TGA, DSC, DMA, and TMA. The thermal data are listed in Table 2. Figure 8 shows the thermal behaviors of the PI films via the TGA measurements. All the PI films exhibited good thermal stability before 500 °C in nitrogen. The PI-ref film exhibited a bit higher initial thermal decomposition temperature than those of the NDA-PI films. The 5% weight loss temperature (*T*_5%_) of PI-ref is 581 °C, which is 65.3 °C higher than that of the PI-a (PMDA-NDA). The introduction of benzimidazole units increased the *T*_5%_ values of the NDA-PI films. PI-d shows a *T*_5%_ value of 541.7 °C, which is 26.0 °C higher than that of the PI-a. All the PI films left nearly 60 wt % of their original weights at 730 °C.

**Figure 9:**
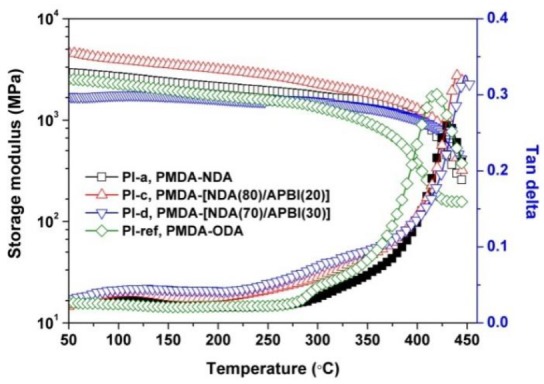
Dynamic mechanical analysis (DMA) curves of PI films.

The glass transition behaviors of the PI films at elevated temperatures were investigated by DSC and DMA measurements. As expected, no clear glass transition was detected during the DSC studies from room temperature to 400 °C due to the insensitivity of such rigid PI films to the DSC measurements [30]. More detailed information was obtained from the DMA measurements. Figure 9 illustrates the mechanical relaxation spectra of PI films. The change of storage modulus and tan delta of the PI films with the increasing temperatures were recorded in the figure. All the PI films maintained good stability with the nearly constant storage modulus before 350 °C, after which the storage modulus began to decrease. When it reached 450 °C degrees, the modulus of the PI films dropped no more than two orders of magnitude of their initial modulus. The *T*_g_ values of the PI films were recognized as the peaks of the tan delta plots. According to the data shown in Table 2, the *T*_g_ values of the PI films increased with the order of PI-ref < PI-a < PI-b < PI-c < PI-d. Apparently, the introduction of benzimidazole units increased the thermal stability of the NDA-PI films. 

**Figure 10:**
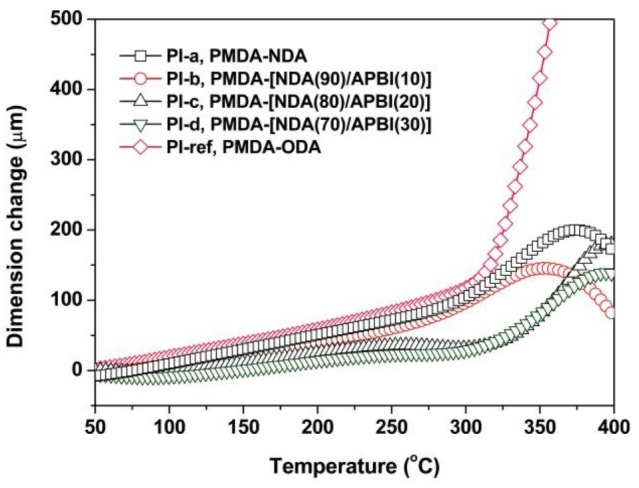
Thermomechanical analysis (TMA) curves of PI films.

At last, the dimensional stability of the PI films at elevated temperatures were investigated by the TMA measurements and the CTE values of the PI films in the temperature range of 50–250 °C were recorded, as shown in Figure 10 and Table 2. Benzimidazole units have been widely incorporated into the molecular structures of PI films either for decreasing the CTE values of the films [31,32], increasing the modulus of the films [33], increasing the *T*_g_ values of the films [34], or increasing the bonding strength with the copper foil [35] due to the strong intermolecular interactions resulting from the formation of the hydrogen bonding for the benzimidazole-containing PI films [36]. It can be deduced from Figure 10 that the introduction of benzimidazole units apparently decreased the dimensional change of the PI films. PI-d with the highest benzimidazole contents showed a CTE value of 8.9 × 10^−6^/K, which is obviously lower than those of the PI-a and PI-ref films. In addition, PI-b exhibited a CTE value of 17.2 × 10^−6^/K, which is very close to that of the copper foil (CTE = ~17 × 10^−6^/K). Thus, it could be prospected that the black FCCLs fabricated with the PI-b as the substrate film and copper foil as the conductor might show enhanced reliability in the practical applications. 

It is worthy of noticing that all the NDA-PI films exhibited significant peaks of dimensional change in the temperature range of 350–400 °C. That is to say, the NDA-PI films exhibited the dimensional change trend of first expanding and then shrinking during the heating process, while the PI-ref film showed the single expanding behavior. The reason for this phenomenon is still uncertain, which might be due to the possible slight crosslinking reaction of the chemically active imino (−NH−) groups in the molecular structures of the NDA-PI films at elevated temperatures. This high-temperature shrinking feature for the current PI films is interesting and might be meritorious for their practical applications because this shrinkage makes it possible to prepare the PI films with the CTE value of 0 in theory. We will continue to study this in detail in our following work.

## 4. Conclusions

A series of intrinsically black PI films with excellent blackness and opaqueness and excellent thermal and dimensional stability have been designed and developed in the current work. The *T*_g_ values of the developed black PI films were higher than 400 °C, and the CTE values were in the range of (8.9–17.2) × 10^−6^/K in the temperature range of 50–250 °C. The optical transmittances of the films at the wavelength of 600 nm were less than 2%. The good comprehensive properties make the functional PI films good candidates for the fabrication of high-performance black FCCLs. 

## Figures and Tables

**Table 1:** Inherent viscosities and molecular weights of poly(amic acid) (PAAs).

PI	[*η*]_inh_^a^ (dL/g)	*M*_n_^b^ (g/mol)	*M*_w_^b^ (g/mol)	PDI ^b^
PI-a	0.98	58,281	129,744	2.23
PI-b	0.86	38,852	72,264	1.86
PI-c	0.73	34,599	59,856	1.73
PI-d	0.71	32,971	54,072	1.64

^a^ Inherent viscosities measured with a 0.5 g/dL PI solution in *N*-methyl-2-pyrrolidone (NMP) at 25 °C; ^b^ *M*_n_: number average molecular weight; *M*_w_: weight average molecular weight; PDI: polydispersity index, PDI = *M*_w_/*M*_n_.

**Table 2:** Optical and thermal properties of PI films.

PI	Optical Properties ^a^						Thermal Properties ^b^			
	Λ (nm)	*T*_600_ (%)	*T*_760_ (%)	*L* ^*^	*a* ^*^	*b* ^*^	*T*_g_ (°C)	*T*_5%_ (°C)	*R*_w730_ (%)	CTE (×10^−6^/K)
PI-a	592	0	36.8	2.20	11.99	3.33	431.6	515.7	59.7	18.8
PI-b	578	0.4	43.1	4.23	23.67	7.26	435.7	545.1	65.3	17.2
PI-c	578	0.5	57.1	5.51	29.31	9.22	440.6	539.4	61.5	11.0
PI-d	561	1.6	58.2	9.58	34.92	16.20	445.5	541.7	64.0	8.9
PI-ref	407	74.6	80.9	88.65	−9.39	79.41	418.8	581.0	61.6	29.5

^a^ λ: Cutoff wavelength; *T*_600_, *T*_760_: Transmittance at the wavelength of 600 nm and 760 nm, respectively; L^*^, a^*^, b^*^, see Section 2.2 Characterizations part. ^b^ *T*_g_: Glass transition temperature; *T*_5%_: Temperatures at 5% weight loss; *R*_w730_: Residual weight ratio at 730 °C in nitrogen; CTE: linear coefficient of thermal expansion in the range of 50–250 °C.
